# Microglial Dysfunction Induced by C9ORF72 Dipeptide Repeat Proteins: Biomarker and Therapeutic Perspectives

**DOI:** 10.3390/ijms27125537

**Published:** 2026-06-18

**Authors:** Niti Sharma, Seong Soo A. An

**Affiliations:** Lab of Ageing-Related Diseases, Bionano Research Institute, Gachon University, 1342 Seongnam-daero, Sujung-gu, Gyeonggi-do, Seongnam-si 461-701, Republic of Korea; nitisharma@gachon.ac.kr

**Keywords:** C9ORF72, dipeptide repeat proteins, microglial dysfunction, amyotrophic lateral sclerosis, frontotemporal dementia, biomarkers, therapeutic strategies

## Abstract

The GGGGCC hexanucleotide repeat expansion (HRE) in *C9ORF72* was recognized as the most common genetic cause of amyotrophic lateral sclerosis (ALS) and frontotemporal dementia (FTD). Repeat-associated non-AUG (RAN) translation of the expanded repeat generated dipeptide repeat proteins (DPRs), which disrupted multiple cellular processes and contributed to neurodegeneration. Emerging evidence indicated that disease pathogenesis involved both gain-of-function (GOF) and loss-of-function (LOF) mechanisms. DPR-mediated GOF toxicity induced ribosomal dysfunction, nucleolar stress, proteostatic impairment, and neuronal injury, whereas *C9ORF72* LOF disrupted lysosomal and autophagic pathways in microglia, impairing the immune homeostasis. Neuronal injury further promoted the release of damage-associated signals that triggered secondary microglial activations and chronic neuroinflammations. This review summarized current knowledge of DPR biology, microglial dysfunction, and their contributions to disease progression in C9ORF72-associated ALS/FTD. Therapeutic strategies targeting repeated RNA, DPR productions, proteostasis, autophagy, and neuroinflammatory pathways were also discussed. In addition, the potentials of fluid biomarkers, including cerebrospinal fluid poly (GP) and blood neurofilament light chain (NfL), for diagnosis, disease monitoring, and therapeutic assessment were shown. Together, these findings provided important insights into disease mechanisms and potential avenues for improved clinical management.

## 1. Introduction

The *C9ORF72* gene, widely expressed in the central nervous system with a role in vesicle trafficking and autophagy, was identified as a key player in neurodegeneration [1]. It was demonstrated that a large GGGGCC hexanucleotide repeat expansion (HRE) in the intronic region of the gene was the main cause in patients with amyotrophic lateral sclerosis (ALS) or frontotemporal dementia (FTD) [2]. HRE was responsible for as many as 40% of familial cases and 5-10% of sporadic cases, and, interestingly, it was more prevalent among Europeans [3]. In addition, a weak association was noticed between *C9ORF72* HRE and other neurodegenerative disorders (NDs), including Parkinson’s disease (PD), progressive supranuclear palsy (PSP), ataxia, corticobasal syndrome (CBS), Huntington’s disease (HD), Creutzfeldt-Jakob disease (CJD), and even Alzheimer’s disease (AD) [4,5].

From a mechanistic perspective, HRE contributed to disease through multiple overlapping pathways [5]. HRE was transcribed in both directions, giving rise to repetitive sense and antisense RNAs that accumulated as nuclear RNA foci. Despite its origination in a non-coding region of *C9ORF72*, these RNAs were still translated through an unconventional process known as repeat-associated non-ATG (RAN) translation. This resulted in the production of five distinct dipeptide repeat proteins (DPRs), poly-glycine-alanine (poly-GA), poly-glycine-proline (poly-GP), poly-glycine-arginine (poly-GR), poly-proline-alanine (poly-PA), or poly-proline-arginine (poly-PR), which presented cellular toxicity [6,7]. The DPRs derived from C9ORF72 repeats tended to have characteristics of intrinsically disordered proteins (IDPs) and, hence, could behave abnormally in terms of interaction with various cell components and biomolecular condensates. The DPRs not only aggregate, but also altered liquid–liquid phase separation (LLPS), forming sticky and very stable structures [8]. This property enabled DPRs, including those enriched with arginine (poly-GR and poly-PR), to disturb RNA processing, ribosomes’ function, nucleolus stability, and stress responses [9,10,11]. As a result, the normal organization of the cell became disrupted, causing important interacting proteins to become trapped or misplaced [12]. Whereas proteins with defined structure affected one particular aspect of cellular functioning, disordered DPRs were capable of disrupting several processes within a cell at once, thus increasing its susceptibility to damage [10].

The disease mechanisms of *C9ORF72* expansions resulted from loss-of-function (LOF; haploinsufficiency) of the C9ORF72 protein and toxic gain-of-function from sense and antisense C9ORF72 repeat RNA or from DPRs [5]. Cumulatively, these effects disturbed key cellular processes, causing significant changes in dendritic morphologies, which were complemented by a higher susceptibility of the neurons to glutamate-induced excitotoxicity [13]. However, these changes were not confined to neurons alone. Increasing evidence showed that C9ORF72-related pathologies were also observed in other cell types, especially microglia [14]. Since *C9ORF72* was highly expressed in microglia, its loss disrupted the normal lysosomal function and altered the immune signaling, predisposing microglia to an inflammatory phenotype [15,16]. At the same time, toxic RNA species and DPRs generated in neurons promoted neuronal stress and injury. The resulting release of extracellular DPRs, misfolded proteins, and damage-associated molecular patterns (DAMPs) subsequently activated neighboring microglia [17,18]. Taken together, this created a harmful cycle where overactive microglia began to remove synapses excessively and sustained the inflammation and, in turn, worsened neuronal damage and sped up disease progression [19].

In this review, the immune aspects of the disease were reviewed, along with a particular emphasis on the role of microglia. Recent advances in *C9ORF72* HRE were discussed to provide a better, comprehensive understanding of the cellular contributions to disease progression. In particular, the association between the excessive activation of microglia and the enhanced detrimental impacts of DPRs was investigated in depth, particularly the facilitated responses to DNA-binding protein 43 (TDP-43) pathology. Understanding these relationships would enable us to suggest a few therapeutic options, such as interference with interferon signaling and enhancing lysosome functioning [20]. In addition, emerging fluid biomarkers, including CSF poly (GP) and blood neurofilament light chain (NfL), may support earlier diagnosis and therapeutic monitoring in C9ORF72-associated ALS/FTD.

## 2. Microglial Dysfunction in C9ORF72 Disease

**Figure 1:**
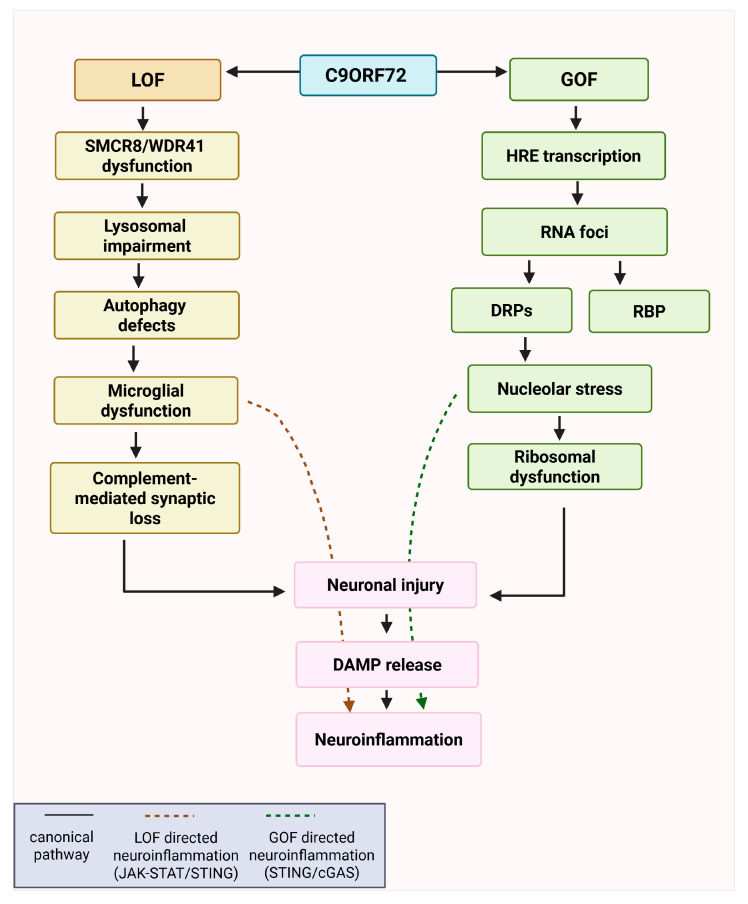
Distinct loss-of-function and gain-of-function mechanisms in C9ORF72-associated ALS/FTD. The *C9ORF72* HRE contributed to disease through both gain-of-function (GOF) and loss-of-function (LOF) mechanisms. In the GOF pathway, repeat-containing RNA formed the sense and antisense RNA foci and related to repeat-associated non-AUG (RAN) translation, generating toxic dipeptide repeat proteins (DPRs). DPRs induced nucleolar stress, impaired RNA metabolism and protein translation, activated the innate immune signaling, and promoted neuronal injury. In the LOF pathway, reduced *C9ORF72* expression disrupted the C9ORF72/SMCR8/WDR41 complex, impairing lysosomal function, autophagy, and microglial homeostasis. Dysfunctional microglia exhibited the impaired phagocytosis and enhanced inflammatory responses, contributing to synaptic loss and neuroinflammation. Neuronal injury further amplified the inflammation through the release of damage-associated molecular patterns (DAMPs), creating a self-reinforcing cycle of neurodegeneration. Dashed lines indicated pathways that may promote neuroinflammation independently of the neuronal injury-DAMP cascade. Created in Biorender.com.

Although *C9ORF72*-related neurodegeneration was often described in terms of RNA foci, DPRs, mislocalization of TDP-43, and reduced protein levels [6,21], microglial dysfunction had also emerged as an important contributor to disease pathogenesis. *C9ORF72* was highly expressed in microglia and had key roles in lysosomal function, autophagy, and membrane trafficking. *C9ORF72* HRE-associated neurodegeneration was driven by both cell-autonomous and non-cell-autonomous mechanisms [22,23,24]. Notably, *C9ORF72* LOF primarily disrupted microglial homeostasis, whereas gain-of-function (GOF) mechanisms driven by repeat RNA and DPRs primarily caused neuronal injury and subsequent inflammatory responses. In microglia, *C9ORF72* LOF disrupted the Smith-Magenis syndrome chromosome region candidate 8 (SMCR8) and the WD repeat domain 41 (WDR41) (C9ORF72/SMCR8/WDR41) complex, impairing endo-lysosomal function and reducing phagocytic capacity while increasing inflammatory sensitivity [15,25]. In parallel, neurons underwent toxic GOF injury mediated by repeat RNA and DPRs, resulting in ribosomal dysfunction, proteostatic stress, and neurodegeneration [26,27]. Consequently, the prominent neuroinflammation observed at later disease stages was largely considered a secondary response to ongoing neuronal injury. As damaged neurons release misfolded proteins, extracellular DPRs, and DAMPs, microglia become activated and amplify inflammatory signaling [28]. The resulting neuroinflammatory response was intensified by the intrinsic vulnerability of C9ORF72-deficient microglia [22] (Figure 1).

### 2.1. Microglial Activation and Neuronal Injury

Activated microglia contributed directly to synaptic loss and neuronal stress mainly from the complement-mediated classical pathway (C1q and C3), in which synapses were tagged and phagocytized by microglia [29]. In C9ORF72-related diseases, it became part of a harmful cycle in which microglial dysfunctions amplified the neuronal injury rather than resolving it. Supporting this view, studies revealed that loss of *C9ORF72* directly redesigned the microglial transcriptome, driving these cells toward activated and interferon-responsive states [30]. Bulk microglia RNA sequencing showed the decreased ARM (activated response microglia) and increases in IRM (interferon response microglia) markers and cytokines in aged *C9orf72*^−/−^ mice [19]. Functionally, C9ORF72-deficient microglia indicated impaired phagocytosis and lysosomal dysfunction, which caused the excessive complement-mediated synaptic pruning, resulting in synapse loss and neuronal deficits [29]. Although early reports suggested minimal neurological effects [15], recent studies in aged *C9orf72^−/−^* mice revealed abnormal neuronal morphology, increased complement-mediated synaptic loss, and deficits in learning and memory [29]. Loss of *C9ORF72* in neurons caused the altered dendritic arborization, reduced synaptic density, abnormal neuronal morphology, and increased vulnerability to glutamate excitotoxicity independent of DPR-mediated toxicity [13]. Similar synaptic loss was observed in the 5XFAD model with C9ORF72-deficient microglia, indicating a direct, cell-autonomous role for this gene in maintaining synaptic integrity [29]. These synaptic deficits were also observed in C9orf72 knockout models lacking DPR pathology, suggesting that microglial C9orf72 loss alone contributed to impaired synaptic integrity [29]. Furthermore, C9ORF72 mutant microglia from ALS patients showed the increased activations of immune-related pathways, particularly after inflammatory priming along with elevated release of factors, such as matrix metalloproteinase-9 (MMP-9) [28]. These activated microglia became toxic to nearby motor neurons, which could be reduced by inhibiting MMP-9. In addition, dipeptidyl peptidase-4 (DPP-4) was identified as a marker of this dysregulated microglial state [28]. Hence, the altered microglial signaling could directly cause the neuronal damage in C9ORF72-associated diseases.

### 2.2. Lysosomal Dysfunction and Innate Immune Activation

At the molecular level, C9ORF72 functioned as part of a three-protein unit with SMCR8 and WDR41 that activated Rab GTPases and intracellular trafficking [31,32]. In addition, the complex could regulate Unc-51-like autophagy-activating kinase 1 (ULK1)-dependent autophagy initiation, linking C9ORF72 to early cellular clearance pathways [33,34]. Disruption of this system would impair vesicle transport and lysosomal functions [35]. Similarly, loss of C9ORF72 disrupted the autophagy-lysosomal and immune pathways, leading to impaired clearance and toxic DPR buildup [36,37]. On the other hand, C9ORF72 deficiency also resulted in functional decline in lysosomal capacity with reduced activity of key hydrolases, such as cathepsin D, and a diminished ability to degrade internalized cargo, resulting in the accumulations of cellular waste [38].

Prominently, the C9ORF72/SMCR8 complex played a role in maintaining lysosomal integrity by supporting endosomal sorting complexes, required for transport (ESCRT)-mediated membrane repair [16]. In its absence, the damaged lysosomes were not repaired efficiently and were accumulated instead, as indicated by increased galectin-3 (Gal3) labeling, particularly in aged or chronically activated microglia [39]. Consistent with this, C9ORF72 deficiency was associated with widespread lysosomal dysfunction, reduced activation of the mechanistic target of rapamycin complex 1 (mTORC1), and impaired autophagic flux, as observed in both knockout models and patient-derived cells [38,40]. Mechanistically, this phenotype appeared to be associated with abnormal regulation of RAB8A. In microglia lacking C9ORF72, leucine-rich repeat kinase 2 (LRRK2) promoted excessive phosphorylation of RAB8A, disrupting its recruitment to damaged lysosomes. Consequently, RAB8A could not effectively contribute to ESCRT-dependent membrane repair, leading to persistent lysosomal damage and reduced restoration of lysosomal integrity [16].

In addition, loss of C9ORF72 reduced the degradation of the stimulator of interferon gene (STING) protein, leading to elevated type I interferon (IFN) signaling in both mouse models and ALS/FTD patients [30]. This indicated that lysosomes were not only involved in waste clearance but also participated as key regulators of immune signaling termination [41]. In parallel, the produced DPRs from repeat expansion could further stimulate innate immune responses through activating NOD-like receptor protein-3 (NLRP3) inflammasome and increasing interleukin-1 beta (IL-1β) productions [41,42]. The accumulations of repeat RNA and related dsRNA species may also cause sustained interferon signaling [43]. This chronic immune activation resulted in excessive removals of excitatory synapses (vesicular glutamate transporter 1: vGLUT1 and postsynaptic density protein 95: PSD95) and disrupted neural circuits, leading to learning and memory deficits [29]. Lysosomal damages and waste buildup drove Janus kinase/signal transducers and activators of transcription (JAK/STAT)-mediated inflammation with increased necrosis factor alpha (TNF-α), IL-6, and IL-1β [44]. Consistent with this, recent research showed that C9ORF72 deficiency also activated the peripheral immune system, as detected by increased cytotoxic T lymphocyte (CD8^+^ T cell) responses and major histocompatibility complex class I (MHC-I) expressions, which further augmented the disease progressions [45]. Thus, *C9ORF72* mutations contributed to a positive feedback mechanism, whereby lysosomal dysfunction enhanced immune responses and subsequent inflammations [36].

### 2.3. DPR Production and Microglial Stress

The expanded HRE repeats in the *C9ORF72* gene produced toxic DPRs through RAN translation [31]. Of those, arginine-rich (poly-GR) was extremely toxic, as it disrupted protein synthesis by slowing translation elongation and causing ribosome stalling [27,42]. The ribosomal arrest products could not be adequately removed due to the absence of lysine residues in DPRs, making them resistant to ribosome-associated quality control (RQC) [46]. DPRs escape RQC due to limited ubiquitination sites, causing accumulations and protein homeostasis imbalance. As a result, DPRs were aggregated within cells and interfered with main protein clearance systems, including proteasome function and autophagy pathways [36]. Furthermore, DPR productions were also influenced by the translational machinery, regulated by ribosomal protein RPL38, the “gatekeeper,” which maintained the start-codon fidelity and reading frame selection. With reduced RPL38 levels, the availability of the 60S ribosomal subunit declined, affecting AUG-initiated translation, permitting near-cognate initiation [47,48]. This change promoted the RAN translation through non-canonical frames and enhanced the generation of specific DPRs, like poly-GR, initiating their toxicity [48,49]. Ribo-toxic stresses were observed with ribosomal collisions, which was triggered by the signaling pathway through sterile alpha motif- and leucine zipper-containing kinase alpha (ZAKα)/p38 MAP kinase (p38 MAPK) signaling, ultimately promoting neuronal death [27].

Additionally, accumulation of PR-rich DPRs in the nucleolus caused cellular stresses, resulting in increased p53 [50,51]. This activated the cell death pathways, including caspase-3-dependent neuronal loss [51]. Reducing p53 activity was shown to decrease DPR toxicity, emphasizing its role in disease progression. These toxic processes disrupted the neuronal homeostasis and survival, thereby contributing directly to neurodegeneration. The repeat expansion increased this complexity. This microglial activation was largely secondary to neuronal injury rather than a direct effect of DPRs on microglia. DPR-induced neuronal stress promoted the release of DAMPs, including ATP, high mobility group box protein 1 (HMGB1), misfolded proteins, and dsRNA, which subsequently activated neighboring microglia and amplified inflammatory signaling [52]. These signals included damage-associated molecules and misfolded proteins that triggered microglial innate immune responses. These processes transformed microglia into disease-associated microglia (DAM) [53,54]. DAMs have enhanced phagocytic function, dysregulated metabolism, and excessive secretion of cytokines and inflammatory factors, like tumor necrosis factor-alpha (TNF-α), interleukin-1 beta (IL-1β), and IL-6 [55], leading to neuroinflammation. The constantly active microglia affect synaptic homeostasis by inducing synaptic pruning via activation of the complement cascade, which leads to neuronal dysfunction and degeneration [56]. Furthermore, the inability of C9ORF72-deficient microglia to clear aggregated proteins and other debris contributes to continuous inflammation. Consistent with this, HRE expression in microglia had minimal effects on viability and inflammatory signaling, suggesting that neuronal distress signals primarily drove microglial activation [57].

Continuous immune stimulation caused microglia to be locked in an irreversible disease state. This was one of the primary sources of neurotoxicity, which had caused neuronal cells to become more sensitive to any form of stress, increasing the chronic neurodegeneration [58]. In addition, the buildup of DPRs caused endoplasmic reticulum (ER) stress and altered the nucleolus functions, adding to the cellular stress signals leading to the stimulation of microglia [51]. This indicated that microglia were impacted not only by the failure of the C9ORF72 protein but also by the neurotoxic environment due to the mutation [41]. Hence, these accumulated alterations collectively increase neurotoxicity, with early microglial activation gradually shifting toward persistent disease-associated inflammatory states during disease progression.

### 2.4. Microglia and TDP-43 Pathology

A key feature of ALS and FTD was the mislocalization and aggregation of TDP-43 [59,60,61]. Microglial dysfunction also appeared to influence the persistence and spread of TDP-43 pathology. Studies suggested that TDP-43 aggregation could be increased through multiple pathways, including stress granule-associated processes, as well as independent mechanisms [62,63]. When microglia failed to clear toxic proteins efficiently, these materials could be accumulated in the extracellular space, while the sustained inflammatory signals would further increase neuronal vulnerability to protein misprocessing [63]. Supporting this, findings from progranulin deficiency models revealed that microglia could shift into a disease-associated state and drove TDP-43 aggregation and neuronal damage directly [63]. This effect was mediated through complement pathways, where blocking key components, such as C1q and C3, reduced the microglial toxicity and protected neurons [63]. In addition, TDP-43 aggregation in neurons triggered microglial activation through damage signals (ATP, HMGB1, misfolded proteins, and dsRNA), creating a feed-forward loop that amplified neuroinflammations and pathology [64]. Taken together, these results suggested a central role for microglia by promoting both the spread and amplifications of TDP-43 pathology.

These findings suggested that C9ORF72-related disease was not driven by neurons alone. It involved a wider disruption in how different cell types interacted within the brain. As microglia lost their ability to maintain balance, they began to contribute to synaptic loss, inflammation, and the spread of pathology. Therefore, targeting lysosomal function, immune signaling, and cellular trafficking may help to slow disease progression, providing other neuron-focused approaches.

## 3. Therapeutic Approaches Targeting Microglia in C9ORF72-ALS/FTD

Microglial dysfunction in C9ORF72-ALS/FTD was mostly driven by C9ORF72 haploinsufficiency, which impaired lysosomal function and autophagy, leading to increased inflammations in contributing to neurodegeneration. In parallel, neuronal toxicity further amplified these effects, emphasizing a combined mechanism of disease progressions. Therefore, current therapeutic strategies should focus on restoring microglial homeostasis rather than broadly suppressing inflammations [65].

At the cellular level, C9ORF72-deficient microglia-like cells (hiPSC-derived) showed impaired phagocytic capacity and increased cytokine levels (IL-6 and IL-1β) [22]. These defects were closely associated with the disruption of the C9ORF72/SMCR8 complex, which played a key role in maintaining cellular proteostasis [66,67]. Pharmacological activation of autophagy by rapamycin partially seemed to rescue these abnormalities, while restoration of *C9ORF72* function or increased SMCR8 expressions improved the lysosomal function and cellular homeostasis [66]. Following these intrinsic alterations, another strategy was to limit microglia-derived toxicity. In C9ORF72 deficiency, several inflammatory cytokines and MMPs were released, which contributed to neuronal damage. Specifically, *C9ORF72* HRE mutant microglia displayed elevated MMP-9 expressions and promoted non-cell-autonomous neurotoxicity [28]. Use of an MMP-9 inhibitor reduced this neurotoxic effect, even under inflammatory conditions, supporting its role as a therapeutic target.

In addition to cell-intrinsic mechanisms, immune signaling pathways further reshaped the microglial activation. C9ORF72-ALS patients lacking *C9ORF72* in myeloid cells showed signs of increased inflammations, mediated in part by IL-17A signaling and high levels of CD80 in microglia. Inhibition of IL-17A through neutralizing antibodies lowered the neuroinflammation in experimental animal models, indicating a possible treatment strategy targeting microglia [68]. Apart from autonomous cellular effects, other extracellular influences may affect disease development. *C9orf72*^-/-^ mice experienced intense inflammation, which was associated with gut microbiota-generated glycogen, causing microglia activations. Treatment with oral α-amylase (glycogen hydrolyzing enzyme) seemed to decrease inflammation and to extend the lifespan of the mice [69].

At the molecular level, several therapeutic approaches were involved upstream by reducing neuronal stress signals that drove microglial activation. The development of antisense oligonucleotides (ASOs) was considered an interesting direction of targeted therapy for *C9ORF72* mutations, as their use allowed reducing the concentration of downstream toxic RNA and proteins [70,71]. ASOs reversed the key cellular abnormalities and improved neuronal function in preclinical models [72]. In this context, compounds BIIB078 and WVE-004 were evaluated in clinical trials, but the outcomes were modest [73,74]. A newer candidate, afinersen (ASO5-2), demonstrated early promise by reducing poly(GP) levels and repeat-containing RNA in a pilot study [75]. In addition, RNA interference (RNAi) therapy using siRNA and artificial microRNA (miRNA) was applied for gene silencing. The siRNAs directed at the repeat sequence and other transcription regulators, like SPT4, diminished RNA foci and DPR formation in cell cultures and animal models [76,77]. Next, the delivery of artificial miRNAs using adeno-associated vector serotype 5 (AAV5) allowed the selective repressions of mutant *C9ORF72* RNA without disrupting wild-type gene expression [78]. In addition to reducing DPR production, clearance strategies of existing DPR aggregates were also explored and revealed their potentials. The use of poly (GA)-specific antibodies reduced the DPR aggregations, limited seeding activity, and improved neuroinflammations and survivals in preclinical models [79,80]. In addition, enhancing DPR clearance through proteostasis pathways, such as overexpression of the small heat shock protein HSPB8, also reduced the DPR levels through autophagy [81].

Another complementary approach was to enhance lysosomal function through progranulin (PGRN) [82]. Both *C9ORF72* and *PGRN* regulated similar lysosomal pathways in microglia, and their disruption led to impaired clearance and increased inflammation [83]. Progranulin-targeting approaches included AAV9 gene therapy strategies, such as PR006, which delivered the *GRN* gene to restore intracellular progranulin. Antibody-based approaches, such as AL001 (latozinemab), increased the extracellular *PGRN* levels by blocking its degradation. While both strategies elevated *PGRN* successfully, clinical benefit remained uncertain, as antibody-based therapy [84] and gene therapy [85] did not demonstrate efficacy in late-stage trials. Another AAV9-based therapy, AVB-101, is currently being evaluated in the ongoing ASPIRE-FTD Phase 1/2 trial [86]. Early results indicated that the treatment was well-tolerated without serious adverse events [87]. Despite promising preclinical results, several candidates, including BIIB078, WVE-004, AL001, and PR006, failed to show meaningful clinical benefit in late-stage trials. Possible reasons include treatment initiation after substantial neurodegeneration, insufficient impact of biomarker changes on established downstream pathology, clinical and biological heterogeneity among C9ORF72-associated ALS/FTD patients, and challenges in achieving adequate CNS target engagement. These findings stressed the need for earlier intervention, improved biomarker-guided patient stratification, and therapies targeting multiple disease mechanisms in future trials.

Finally, small-molecule approaches targeting upstream disease mechanisms were emerging. Compounds, such as metformin, could reduce the repeat-associated non-AUG (RAN) translation and lowered DPR productions in preclinical models [88], and C9ALS-FTD was under clinical trials [89]. The investigational oral drug, TPN-101, modulating inflammation and DNA damages from the repeat expansions by inhibiting LINE-1 reverse transcriptase, showed promising results in Phase 2 clinical trials [90]. These approaches addressed early pathogenic processes and therapeutic targets by reducing microglial activation indirectly and limiting neuronal stress. Overall, the most promising direction was a combined approach that restored the intrinsic microglial function while also reducing external stress signals from affected neurons (Table 1).

## 4. Fluid Biomarkers and Diagnostic Potential of C9ORF72-Related DPRs

The diagnosis of the *C9ORF72* mutation was mainly done through genetic testing, which can identify the GGGGCC hexanucleotide repeat expansion within the *C9ORF72* gene using repeat expansion molecular techniques [93,94]. In addition to genetic diagnosis, DPRs had drawn significant interest as promising fluid biomarkers in ALS/FTD. Among various DPRs, poly (GP) was the most favorable candidate as a diagnostic biomarker due to its solubility and detection capability in CSF. Moreover, their CSF levels correlated with the amount of repeat-containing RNA in the brain and were detectable even before symptom onset [95]. High levels of CSF poly (GP) were reported in symptomatic and presymptomatic patients with *C9ORF72* HREs using highly sensitive immunoassays, such as Simoa (single molecule array) [95,96,97,98]. Other toxic DPRs, particularly poly (GR) and poly (GA), were also detected in CSF by the MDS (mesoscale discovery) assay, but their level did not reflect disease severity, limiting their value as markers of progression [99]. However, their levels rapidly decreased after ASO treatment, suggesting they may still be useful for monitoring whether a therapy is effectively targeting toxic *C9ORF72* repeat transcripts [99].

It was noted that the different types of DPRs might have different pathogenic and diagnostic implications [98]. Among them, arginine-rich DPRs like poly (GR) and poly (PR) were toxic in cell studies and experimental models, but the specific mechanism behind their increased toxicity was not yet known [100,101,102]. The high arginine composition of these proteins may make them prone to modification of arginine methylation by methyltransferase enzymes. This involved the addition of a methyl or dimethyl group to the arginine side chain [98]. In this context, poly (GR) had a greater association with neurodegeneration compared to other types of DPRs, even though they existed in smaller quantities. Poly (GR) pathology occurred more frequently within areas where neuronal death was pronounced and was distinct in cases where the patient had an FTD-motor neuron disease (FTD-MND) phenotype [98]. Also, post-translational modifications of the DPRs, like the asymmetric dimethylarginine (aDMA), were co-localized with the poly (GR) inclusions, further contributing to disease development [98]. The implications for this would be that certain DPR profiles and their modifications could play a role in disease stratification and management for C9ORF72-linked ALS/FTD patients.

Contrary to DPR’s detection in CSF, their identification was difficult in plasma, because they accumulated mainly inside neurons and did not efficiently cross the blood–brain barrier (BBB). As a result, their levels in the periphery were extremely low [18,103]. Since direct plasma detection of DPRs was challenging, biomarkers such as NfL were commonly used in blood to assess neurodegeneration and disease progression in ALS/FTD [104]. Although NfL was not specific to *C9ORF72* mutations, elevated levels strongly correlated with neuronal injury and disease severity [104]. In addition, inflammatory mediators associated with microglial activation and affected proteins to lysosomal dysfunction may serve as complementary biomarkers, reflecting the immune and cellular homeostatic disturbances observed in C9ORF72-associated ALS/FTD [105]. In short, the use of genetic screening combined with fluid biomarkers may help in early diagnosis and better treatment of ALS/FTD associated with *C9ORF72* mutations.

## 5. Conclusions

The pathogenesis of ALS/FTD associated with *C9ORF72* mutations was driven by the combined effects of DPR-mediated toxicity and C9ORF72 haploinsufficiency. DPRs exhibited IDP-like characteristics, enabling them to disrupt multiple cellular pathways, including RNA metabolism, translation, and stress responses. DPRs primarily exerted toxic gain-of-function effects in neurons, causing ribosomal dysfunction, nucleolar stress, proteostatic failure, and neuronal injury. The resulting release of DAMPs and other stress signals promoted the secondary microglial activation. In parallel, C9ORF72 haploinsufficiency directly impaired the lysosomal and autophagic pathways in microglia, establishing a cell-autonomous pro-inflammatory state and reducing the clearance of toxic proteins and cellular debris. Thus, IDP-mediated cellular stress combined with dysfunctional regulation of immune function resulted in a self-reinforcing neurodegenerative process. Therefore, effective treatment should address both these aspects of the disease. The approaches that targeted specific features of DPRs while improving the functions of microglial cells are important. At the diagnostic level, fluid biomarkers such as CSF poly (GP) and blood NfL may also help in early detection, monitoring disease progression, and evaluating therapeutic response in C9ORF72-associated ALS/FTD. Despite significant advances, several questions remained to be resolved. The relative contributions of C9ORF72 haploinsufficiency, RNA foci toxicity, and DPR-mediated toxicity to disease initiation and progression remained incompletely understood, and emerging evidence suggested that these mechanisms acted in concert rather than independently. In addition, the interplay between cell-autonomous microglial dysfunction and non-cell-autonomous neuroinflammatory responses to neuronal injury required further investigation. Although several therapeutic approaches revealed promise in experimental models, their long-term efficacy and safety in patients remain to be established. Similarly, while poly (GP) and NfL had advanced the field as biomarkers, additional studies would be needed to improve disease stratification and treatment monitoring. Future research should integrate cell-type-specific mechanisms, robust biomarker development, and targeted therapeutic strategies to advance precision medicine approaches for C9ORF72-associated ALS/FTD.

## Figures and Tables

**Table 1:** Therapeutic strategies modulating microglial dysfunction in C9ORF72-ALS/FTD.

Strategy	Target/ Pathway	Therapies	Mechanism	Microglia Targeting	Evidence	Ref.
Autophagy enhancement	Lysosome/ autophagy	Rapamycin	Restores autophagy and cellular clearance	Direct	Preclinical	[66]
MMP inhibition	MMP-9	MMP-9 inhibitors	Reduces microglia-driven neurotoxicity	Direct	Preclinical	[28]
Immune modulation	IL-17A pathway	Anti IL-17A antibodies	Suppresses pro-inflammatory signaling	Direct	Preclinical	[68]
Gut-immune axis	Microbiome/inflammation	α-amylase	Reduces systemic inflammation affecting microglia	Indirect	Preclinical	[69]
ASOs	Mutant RNA	BIIB078, WVE-004, Afinersen	Reduce RNA foci and DPR production	Indirect	Clinical/ preclinical	[75,91,92]
RNAi	Mutant RNA/SPT4	siRNA, AAV5-miRNA	Suppress mutant transcript Expression	Indirect	Preclinical	[76,77,78]
DPR clearance	DPR aggregates	Anti-poly (GA) antibodies	Remove toxic protein aggregates	Indirect	Preclinical	[79,80]
Proteostasis	Autophagy	HSPB8 overexpression	Enhance DPR clearance via Autophagy	Indirect	Preclinical	[81]
PGRN pathway	Lysosomal function	PR006, AL001, AVB-101	Improve lysosomal activity and inflammation	Indirect	Clinical	[84,85,86,87]
RAN translation inhibition	DPR production	Metformin	Reduce DPR synthesis	Indirect	Preclinical/clinical	[88,89]
DNA damage modulation	LINE-1 RT	TPN-101	Reduce DNA damage and inflammation	Indirect	Clinical	[90]

Note: Direct referred to the modulation of implicated pathways in microglial dysfunction and did not imply microglia-specific targeting. Indirect strategies acted through neuronal or systemic pathways that subsequently influenced microglial function rather than targeting microglia directly. Abbreviations. AAV: Adeno-associated vector; ASO: Anti-sense oligonucleotides; DPR: Dipeptide repeat proteins; HSPB8: Heat shock protein family B member 8; miRNA: Micro RNA; MMP: Matrix metalloproteinase; LINE-1 RT: Long interspersed nuclear element-1 reverse transcriptase PGRN: Progranulin protein; RAN: Repeat-associated non-AUG; RNAi: RNA interference; siRNA: Small interfering RNA; SPT4: Suppressor of Ty 4.

## Data Availability

No new data were created or analyzed in this study. Data sharing is not applicable to this article.
